# A framework for utilizing leaf-associated microbes to achieve conservation and restoration goals

**DOI:** 10.1128/msphere.01082-24

**Published:** 2025-05-16

**Authors:** Jacob A. Heil, Jessica R. Bernardin, Stephanie J. Galla, Leonora S. Bittleston

**Affiliations:** 1 Department of Biological Sciences, Boise State University 166917, Boise, Idaho, USA; E O Lawrence Berkeley National Laboratory, Berkeley, California, USA

**Keywords:** leaf, microbiome, phyllosphere, conservation, restoration, plant health

## Abstract

Plant-associated microbiomes have profound effects on ecosystem functioning and play a role in the success of plants at both small and large scales. As key components of healthy plants and ecosystems, plant microbiomes should be considered in conservation and ecosystem management strategies. Many knowledge gaps and logistical barriers exist that increase the difficulty of employing microbes in conservation; however, some success has been achieved by manipulating the root microbiome and in agricultural contexts. In contrast with the root microbiome, the role of the leaf microbiome in conservation remains largely unexplored. In this perspective, we posit that the leaf microbiome plays an essential role in plant and ecosystem health and should be considered in conservation strategies. We include a framework for approaching leaf microbiome management, including identification of sources of disturbance, identifying mechanisms to address resulting plant stress, types of microbial inoculation to achieve desired outcomes, and co-producing plans of management with interest groups and rights holders.

## PERSPECTIVE

From its foundation, the field of conservation biology has been inherently multidisciplinary, bringing together diverse tools for managing biodiversity amid global anthropogenic change (1). First described by Soulé (2), the field of conservation biology operates to conserve and restore biotic communities, which can involve interdependent species that have coevolved with one another (2). Relatively little attention has been given to the potential role of microbial communities in conservation and their host interactions, despite microbes constituting the majority of biodiversity and cellular biomass on Earth (3) and providing important functional roles to hosts and ecosystems (4, 5). Plant-associated microbes are increasingly utilized in agriculture to promote plant health (6), but their use in conservation is mainly limited to applications of root associates.

Several reviews have provided a call to action for conserving microbial communities and their interactions with hosts and other microbes in the Anthropocene (7–9). This increased attention has been supported by the decreasing cost of high-throughput sequencing efforts for non-model species, including metagenomic and amplicon sequencing efforts to characterize microbial communities (10). Microbiome sequencing efforts have revealed the complexity and heterogeneity of microbial communities across space (11), time (e.g., references 12, 12), and even host tissue types (e.g., references 13, 13). For example, studies of plant microbial communities have revealed distinct communities from all plant compartments (e.g., roots, stems, leaves, or flowers) and microenvironments (14, 15). Research investigating microbial interactions with plants has largely been confined to model or crop species and the root microbiome (16, 17). However, there is an opportunity to better understand non-model and wild plant microbial communities and how this information can be used to foster resilience in the face of significant human-induced environmental changes. In this perspective, we focus on the microbial community from an understudied plant compartment: the leaf microbiome, or phyllosphere.

## IMPORTANCE OF FOLIAR MICROBES AND THE NEED FOR THEIR INCLUSION IN PLANT CONSERVATION

The bacteria, fungi, and other microbes of the phyllosphere have a strong influence on the health of their host plant (18). Their effects range from pathogenic to beneficial, and the specific effect of a microbe can depend on the environment and community structure (18, 19). Due to this complexity, it is imperative to approach plant health from a holistic standpoint that considers interactions between the plant and the microbiomes of their various compartments, otherwise known as a “holobiont.” The effects of foliar microbes on plants have implications for the functioning of whole ecosystems. For example, some notable leaf pathogens are responsible for the mortality of large percentages of plant populations such as white pine blister rust impacting white pine species (20); powdery mildew, a generalist (21); and pine pitch canker disease impacting many *Pinus* species (22). Due to the inevitable exchange of microbes between natural and agricultural systems, foliar pathogens threaten not only ecosystems but also human food security (23). While foliar pathogens have been extensively studied, the positive effects of the leaf microbiome may be just as significant as the negative impacts of these pathogens.

## FUNCTIONS OF THE PHYLLOSPHERE MICROBIOME

Phyllosphere communities can affect individual plant fitness and ecosystem functions (24, 25). On a large scale, phyllosphere communities often play a role in the nutrient cycle through nitrogen fixation (26, 27), decomposition (28), and transformation of nutrients into more plant-available forms (29). On the scale of individual plants, the leaf microbiome can support plant health and fitness by promoting growth (30), increasing biomass and fruit production (31, 32), synthesizing vitamins and amino acids (33), and protecting against pathogens and herbivory (34). The diverse benefits of the phyllosphere community for plant fitness and whole ecosystem functioning suggest that they can be leveraged for achieving plant conservation goals.

Locally, climate change is increasingly disrupting the structure of biotic communities, often to the detriment of endemic species (35). Phyllosphere microbes play a crucial role in helping plants tolerate stress and adapt to changing environmental conditions. They can do this through several processes, including the production of secondary metabolites (36, 37), production of heat shock and antifreeze proteins (38, 39), bioremediation (40), assisting in heavy metal uptake (41), and even altering a plant’s physiological response to changing environmental conditions (42, 43). These examples are evidence of the substantial contributions phyllosphere microbes make to plant stress tolerance and environmental adaptation and highlight how they may be a suitable target to explore when managing plant communities affected by climate change

The application of phyllosphere microbes in plant conservation is challenged by complexity, particularly because the varied and context-dependent nature of microbial functions makes their use complicated. For example, disease mediation in the phyllosphere can occur through several mechanisms, including antagonistic interactions with other microbes, such as antibiotic production (44) or direct competition for resources (45). Another method of protection is induced systemic resistance (ISR), where phyllosphere microbes trigger plant immune responses (46, 47). However, an imbalance in the microbial community, known as phyllosphere dysbiosis, can disrupt these protective functions, making plants more susceptible to diseases and environmental stressors (33). This is one example from one plant stress type. The reality is that there are many types of plant stress, and unique complications arise from different combinations of plant stressors, environmental context, and microbial communities. Refining the methods for any foliar inoculation application will require extensive customization for each situation as these interactions may lead to unpredictable outcomes

## INCORPORATING THE LEAF MICROBIOME IN CONSERVATION

**Fig 1:**
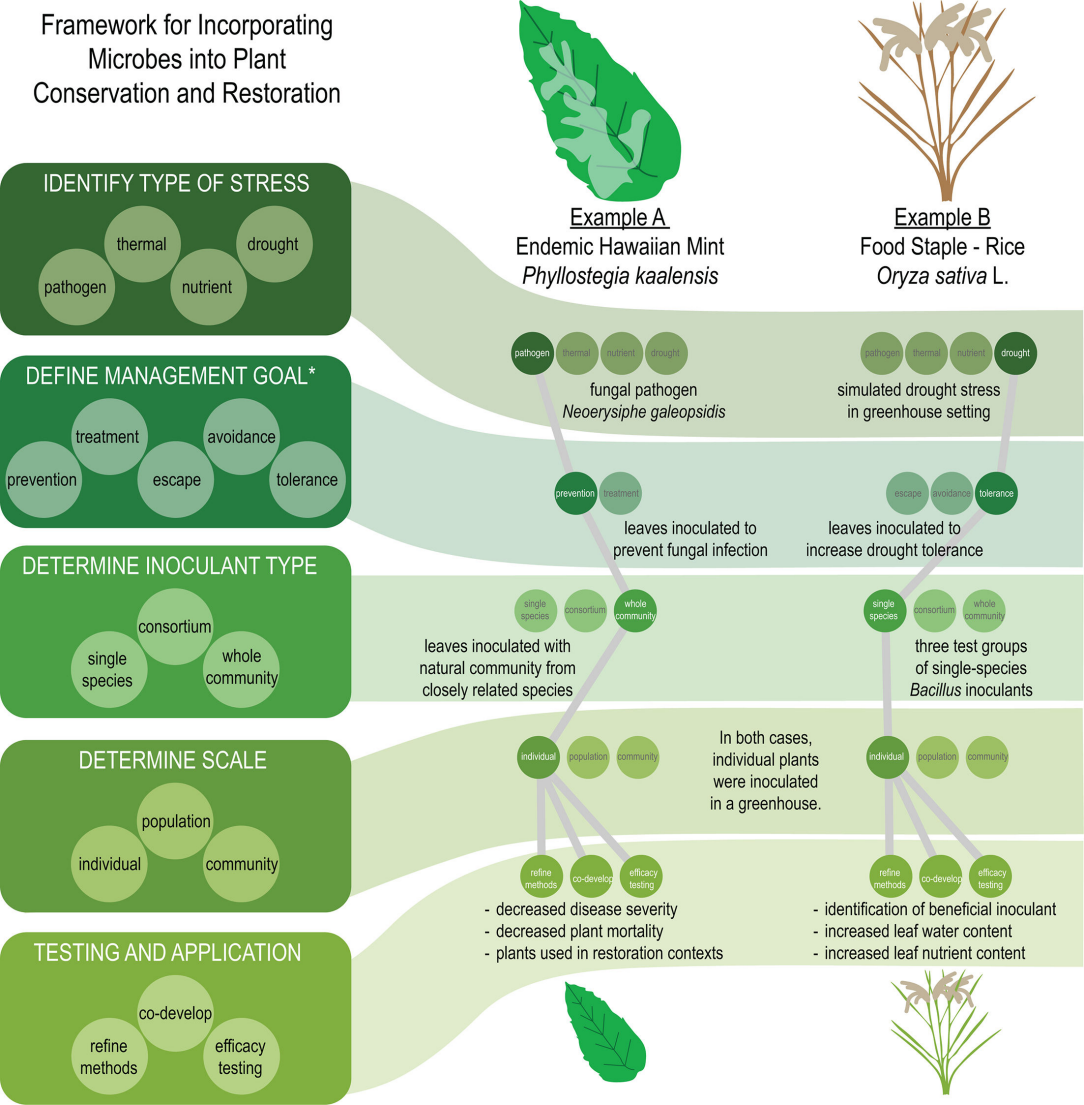
A framework for incorporating leaf microbes in management plans. Our framework includes five steps in developing management plans for the use of foliar microbes as inoculants in the context of management. To demonstrate this framework, we use two real-world examples from references 48 and 49. *Management goals are specific to stress type, e.g., prevention and treatment are management goals for pathogenic stress, but not drought stress.

We created a five-step framework to outline the process by which plant conservation could utilize foliar microbes for management plans (Fig. 1). First, we (1) consider the type of stress that an agent is aiming to remediate (e.g., disease resilience, drought tolerance, and poor plant establishment). After defining the stressor, we can (2) identify a plant management goal. For example, in the case of a plant pathogen, one might aim to prevent plants from being infected with the pathogen (prevention) or to decrease the severity of the pathogen’s effects on the plant (treatment). Leaf microbiome manipulation as a management tool is limited to the outcomes that are within the known functions of leaf-associated microbes. Therefore, we assert that (3) inoculants must be studied, tested, and designed to target these specific functions to achieve the desired results. We anticipate that inoculants may range from single species to whole communities. On one end of the spectrum, a single-species inoculant is likely to be highly targeted, with a specific outcome in mind; conversely, a whole community derived from an environmental sample will likely have less targeted effects and could be used more generally to promote diversity. The scale of application (4) will need to be considered as it can range from individuals to whole plant communities. Finally, we (5) acknowledge that the development of a management plan of this nature will require extensive development and communication with interested parties and rights holders. The complexities of developing inoculants and methods of inoculation are beyond the scope of this perspective, but all strategies will require extensive laboratory testing, ground-truthing, and ethical considerations to develop successful management methods. To demonstrate how this framework fits real-world scenarios, we highlight two types of environmental stressors: plant pathogens and drought.

## AN EXAMPLE OF PLANT PATHOGEN MANAGEMENT

Plant pathogens can range from generalists that attack many hosts to specialists that attack one or few. In both groups, there exist different mechanisms of pathogenicity and therefore pathways to mitigate their damage (50). For example, microbial inoculants on host plant leaves have had some success as biocontrol agents in agriculture (e.g., *Bacillus amyloliquefaciens* can inhibit several pathogenic fungi) (51, 52) but have rarely been used in ecosystem management (53). Management outcomes addressing plant pathogens can be broadly defined as prevention or treatment (Fig. 1). In the context of leaf inoculation, prevention strategies would leverage priority effects by establishing a population of pathogen-antagonists early in the plant’s life or by priming the immune systems of seedlings for future responses to pathogen invasion (54, 55). Treatment strategies would seek to minimize the effects of pathogens by direct antagonism or by promotion of a general community diversity to decrease the abundance of a pathogen (48). To achieve these outcomes, inoculants can range from highly targeted single-species inoculants to broad-spectrum consortiums of pathogen antagonists to inoculation with whole natural communities to promote a highly diverse and competitive environment. Pathogen identity and the inoculant strategy will inform the scale of management, from inoculation of individual seedlings to whole communities of adult plants. A rare example of successful disease management outside of agriculture highlights the elements of this framework (Fig. 1) (48). In this case, powdery mildew (*Neoerysiphe galeopsidis*) was successfully treated in *Phyllostegia kaalensis* by inoculating individuals with a natural whole community from the leaves of a related plant species. This research involved co-production and participation from a number of different groups, including a local arboretum where seedlings were grown, and the Army Natural Resources program and National Science Foundation, which both provided funding as well as contributions from individuals. It should be noted that in this study, powdery mildew remained present in the leaf community. Many pathogens are latent in natural leaf communities, and because of this, whole-community leaf inoculation studies can often yield negative effects on plant health (53). Biocontrol successes using more targeted leaf inoculations can be found in agricultural literature and could be used as examples for future applications in natural ecosystems (Fig. 1) (51, 53).

## AN EXAMPLE OF DROUGHT MANAGEMENT

Regimes of climatic disturbance, such as drought, are rapidly changing and challenging plant health in novel contexts (56). Plant physiological responses to drought are categorized as escape (enhanced growth and reproduction), avoidance (management of internal water conditions), and tolerance (endurance with low water content) (57). The functional roles of foliar microbes can assist plants in these strategies, and management outcomes should target the appropriate response (Fig. 1). For example, foliar plant growth-promoting bacteria can be leveraged to enhance a plant’s ability to escape drought through enhanced growth and reproduction (58). Foliar microbial osmoprotectant production aids plants in drought avoidance by protecting the internal water supply (36, 37). Modification of chlorophyll fluorescence and promotion of gas exchange by foliar microbes can increase the photosynthetic rate and efficiency to promote drought tolerance in plants (59). The specificity of these interactions highlights the necessity of targeted inoculants, and several candidate microbes have already been leveraged for drought resistance in agriculture (60). Less work has been done to establish drought-resistance effects using whole microbial communities; however, xeric plants have shown strong selection in the phyllosphere, suggesting the potential for host selection of these traits in their microbial communities (61). Furthermore, evergreen xeric shrubs (e.g., *Artemisia tridentata*) harbor communities year-round, creating further potential for the evolution of communities of microbial leaf specialists (62, 63). The scale of inoculations (e.g., from individuals to communities) to address drought is context-dependent and should consider the nature of plant interactions in the target ecosystem. Drought affects whole plant communities; therefore, one approach could be to seek generalist microbes to broadcast and promote drought resistance across the entire plant community. However, it may be useful to leverage targeted inoculations and inter- and intraspecific plant interactions in the case of ecosystems where nurse plants promote drought resistance in the whole community (64). Drought resistance promotion in plants via foliar inoculation is currently contained in the realm of agriculture, and the successes in agriculture should be leveraged for natural ecosystem management (60).

## KNOWLEDGE GAPS AND FUTURE DIRECTIONS

Each step in the framework has its own set of knowledge gaps that need to be addressed during the development of foliar inoculation applications for plant conservation. To determine management goals for plants, researchers will need highly specific knowledge of the plant physiological response that they want to elicit from inoculation. For inoculants, little is known about the relative efficacy of single species vs consortium vs whole community inoculants. For scale, many details remain unknown as to the specifics of inoculation, including ideal plant age, time of year for field inoculation, ideal environmental conditions, and the exchange of inoculated microbes between plants in-field. With our proposed framework, knowledge gaps can be more easily addressed in future studies through categorization of details.

## CONCLUSION

Phyllosphere microbes have a robust and barely tapped potential for the management of plant stress in disturbed ecosystems. Foliar inoculants are currently being leveraged in agriculture to promote plant health and achieve management outcomes directly relevant to plant conservation, demonstrating the efficacy of these approaches. We argue that foliar inoculation should be prioritized as a strategy for plant conservation and provide a conceptual framework for implementation.

As with all communities, leaf microbiomes contain organisms that often experience limitations to dispersal and have adapted to local conditions, leading to distinct biogeographical distributions. Human activity threatens endemic populations, and the management discussed in this paper could cause unintended shifts in local microbial species (65). We consider it an ethical imperative to protect endemic microbiomes whenever possible (66, 67) and recommend that all management strategies should begin with locally sourced microbes. Implementation of bacterial treatments for management purposes will require strong associations between researchers and habitat managers. To facilitate these relationships and ensure that bacterial treatments are responsive to local needs, we recommend a co-production approach to research and implementation by incorporating input from scientists, practitioners, and funders throughout the process, using approaches outlined in references 68–70. Research co-production engages researchers and practitioners from the conception of research questions through the implementation of restoration approaches. This approach leverages local knowledge, discovers shared goals (71), enhances benefit-sharing with diverse communities (69), and provides a mechanism to overcome challenges associated with the research implementation gap (72). Because the inoculation approaches described here are novel, we anticipate this approach will lead to more trusted methods, community support, and successful implementation at the appropriate scales.
